# Bridging gut microbiota composition with extended-spectrum beta-lactamase *Enterobacteriales* faecal carriage in critically ill patients (microbe cohort study)

**DOI:** 10.1186/s13613-023-01121-0

**Published:** 2023-04-04

**Authors:** Renaud Prevel, Raphaël Enaud, Arthur Orieux, Adrian Camino, Pierre Sioniac, Fatima M’Zali, Véronique Dubois, Patrick Berger, Alexandre Boyer, Laurence Delhaes, Didier Gruson

**Affiliations:** 1 Medical Intensive Care Unit, CHU Bordeaux, 33000 Bordeaux, France; 2 Centre de Recherche Cardio-Thoracique de Bordeaux Univ Bordeaux Inserm UMR 1045, 33000 Bordeaux, France; 3 CHU Bordeaux, CRCM Pédiatrique, CIC 1401, 33000 Bordeaux, France; 4 Univ Bordeaux UMR 5234 CNRS, 33000 Bordeaux, France; 5 Bacteriology Department, CIC 1401, CHU Bordeaux, 33000 Bordeaux, France; 6 Mycology-Parasitology Department, CIC 1401, CHU Bordeaux, 33000 Bordeaux, France

**Keywords:** Extended-spectrum beta-lactamase, Microbiota, Mycobiota, Intensive care unit, Colonization resistance

## Abstract

**Background:**

The worldwide dissemination of extended spectrum beta-lactamase producing *Enterobacteriales* (ESBL-E) is of major concern. Microbiota may play a role in the host resistance to colonization with ESBL-E, but the underlying mechanisms remain unknown. We aimed to compare the gut microbiota composition between ESBL-producing *E. coli* or *K. pneumoniae* carriers and ESBL-E non-carriers according to the bacterial species.

**Results:**

Among 255 patients included, 11 (4,3%) were colonized with ESBL-producing *E. coli* and 6 (2,4%) with ESBL-producing *K. pneumoniae,* which were compared with age- and sex-matched ESBL-E non carriers. While no significant differences were found between ESBL-producing *E. coli* carriers and non-carriers, gut bacteriobiota α-diversity was decreased in ESBL-*K. pneumoniae* faecal carriers compared both with non-carriers (*p* = 0.05), and with ESBL-producing *E. coli* carriers. The presence of *Sellimonas intestinalis* was associated with the absence of ESBL-producing *E. coli* fecal carriage. *Campylobacter ureolyticus*, *Campylobacter hominis*, bacteria belonging to *Clostridium* cluster XI and *Saccharomyces sp.* were associated with the absence of ESBL-producing *K. pneumoniae* faecal carriage.

**Conclusions:**

The composition of the gut microbiota differs between ESBL-producing *E. coli* and *K. pneumoniae* faecal carriers suggesting that microbial species should be taken into account when investigating the role of gut microbiota in resistance to gut colonization with ESBL-E.

*Trial registration number*: NCT04131569, date of registration: October 18, 2019.

**Supplementary Information:**

The online version contains supplementary material available at 10.1186/s13613-023-01121-0.

## Background

Antimicrobial resistance in both community and healthcare-acquired infections is a major public health concern leading to about 35,000 associated deaths per year in Europe [1] and with 10,000,000 deaths per year worldwide expected in 2050 according to the World Health Organization [2]. Extended spectrum beta-lactamase (ESBL) producing *Enterobacteriales* (ESBL-E) are a major source of both antimicrobial resistance and empirical antimicrobial therapy failure in Europe. Containment measures, including antimicrobial stewardship, have been installed but with limited impact [3]. Consequently, new strategies to contain ESBL-E dissemination are urgently needed.

Gut microbiota is nowadays suggested to be a key player in host resistance to colonization with multi-drug resistant organisms (MDRO), including ESBL-E [4]. This resistance to colonization is mediated both by indirect mechanisms (i.e., the microbiota stimulating host immune defences) but also by direct interactions between microbial species as commensal species can directly suppress intestinal pathogens by competitive exclusion and antimicrobial activities (i.e., type IV secretion systems, production of bacteriocins, …) [4, 5]. A first step into the analysis of gut microbiota composition associated with the resistance to the acquisition of MDRO has identified the presence of *Lactobacillus sp*. to be protective but this study did not specifically assess the type of antimicrobial resistance nor the bacterial species [6]. A step forward has been performed regarding colonization with vancomycin-resistant *Enterococcus faecium* (VRE) allowing the identification of a bacterial consortium restoring colonization resistance to VRE [7]. The modulation of gut microbiota has been proposed as a new strategy to contain MDRO dissemination by re-establishing microbiota-mediated colonization resistance which markedly reduced infections with those antibiotic-resistant bacteria [5].

However, the association of gut microbiota composition and the underlying mechanisms involved in the resistance to colonization with ESBL-E in critically ill patients remains unknown. The impact of the bacterial species carrying the ESBL enzyme, *Escherichia coli* or *Klebsiella pneumoniae* being the most frequently isolated species on clinical samples, is also unknown. The aim of this study is thus to compare the gut bacterial and fungal microbiota (bacteriobiota and mycobiota) composition between patients colonized with ESBL-producing *E. coli* and *K. pneumoniae* and to compare gut microbiota composition between ESBL-E faecal carriers and non-carriers according to the bacterial species carrying the ESBL enzyme.

## Methods

### Patients inclusion and data collection

Every consecutive patient older than 18 years of age admitted to the medical ICU at Bordeaux university hospital from October 2019 to March 2020 was prospectively screened to participate to Microbe study (NCT04131569). ESBL-E faecal carriage was diagnosed by isolation of bacteria from rectal swabs performed within the first 2 h after admission to ICU prior any antimicrobial therapy in ICU on chromID ESBL^®^ culture media (bioMérieux^®^ reference number: 43481) and confirmed by culture with MAST^®^ AMPC & ESBL DETECTION DISCS D68C. Bacterial or fungal species were further identified using MALDI–TOF mass spectrometer, Bruker Biotyper, Bremen, Germany^®^.

Data were prospectively recorded by physicians in charge of the patient by questioning the patients, patients’ family and patients’ general practitioners. Electronic worksheets were completed by two medical intensive care residents. Definitions of comorbidities are available in the Additional information.

### Samples collection and preparation for microbiota analysis

Rectal swabs (Transport Swab VWR, Copan^®^) used for faecal ESBL-E carriage screening at admission were collected and frozen at − 80 °C. DNA extraction was performed by QIAamp^®^ PowerFaecal^®^ Pro DNA kit (QIAgen^®^). A step of mechanical lysis (2 cycles of 30 s at 7,000 rpm on Precellys evolution) was added to the chemical lysis of the kit as previously described [8]. V3–V4 regions of 16SRNA gene and ITS2 loci of rDNA gene were amplified by PCR. Sequencing (2 × 250 bp paired-end) was performed on MiSeq sequencer (Illumina^®^) at the PGTB platform (INRAe, Pierroton, France).

### Bioinformatics analysis

DADA2 pipeline on R software (R foundation for Statistical Computing Vienna, Austria) was used for bioinformatics analyses [9]. DADA2 pipeline was preferred as it allows inter-studies comparison (if identical primers are used for amplification) [9] and is more accurate for mycobiota analysis [10]. We defined bacteriobiota as the bacterial kingdom of the microbiota and mycobiota as the fungal kingdom of the microbiota. Gut bacteriobiota and mycobiota α-diversity was expressed by Shannon index, Simpson index and evenness. This reflects the within sample diversity taking into account the number of different species and their relatives abundances in this sample. Between sample beta-diversity differences (measured using Bray Curtis dissimilarity) were tested using a permutational multivariate ANOVA (PERMANOVA) from “vegan” package with 10,000 permutations, while accounting for individual identity as a covariate. This β-diversity reflects between sample diversity and how similar is the composition of the microbiota between the samples included in the analysis. Gut bacteriobiota and mycobiota α and β diversities were compared thanks to “Phyloseq” package on R software. Linear discriminant analysis (LDA) effect size (LefSe) analysis was performed was performed from microbiomeMarker package. In brief, this analysis first uses the non-parametric factorial Kruskal–Wallis (KW) sum-rank test to detect features with significant differential abundance with respect to the class of interest (ESBL-E facal carriage or not); biological significance is subsequently investigated using a set of pairwise tests among subclasses using the (unpaired) Wilcoxon rank-sum test. As a last step, LEfSe uses Linear Discriminant Analysis to estimate the effect size of each differentially abundant feature [11]. We used mock communities (compositions in the Additional information) and negative controls (three from the DNA extraction step with unloaded swabs and three from the PCR amplification step) to ensure the sequencing quality. Comparison of β-diversity between negative control, mock community and samples are available in Additional file 1: Figs. S1 and S2 for bacteriobiota and mycobiota respectively. The final average read counts were 66,434 (standard deviation ± 17,634) for 1285 bacterial amplicon sequence variants (ASVs) and 3,647 (standard deviation ± 1267) for 361 fungal ASVs. The 16S rRNA gene and ITS2 sequences have been submitted to the European Nucleotide Archive (Accession N◦ Accession N◦ ERP134947). Statistical analysis was performed with the R studio program (version 1.3.1056 for Windows^™^); correction for multiple-testing was performed using the Benjamini–Hochberg false discovery rate (FDR) procedure, a *p* value or FDR adjusted *p* value equal to or less than 0.05 was considered statistically significant.

### Statistical analysis

No statistical sample size calculation was performed a priori, and sample size was equal to the number of patients admitted to ICU during the study period. As ESBL-E fecal carriers, especially these colonized with ESBL-producing *K. pneumoniae*, are known to have more comorbidities than ESBL-E non fecal carriers [12, 13], control patients were selected as consecutive patients matched on sex and age ± 5 years but not on comorbidities and severity scores.

Quantitative variables are presented as median and interquartile range (IQR) and compared by use of the Mann–Whitney Wilcoxon rank-sum test. Categorical variables are expressed as number of patients (percentage) and compared by mean of the χ2 or Fisher tests. All statistical tests were two-tailed and statistical significance was defined as *p* < 0.05. Statistical analyses were assessed by the R 3.6.0 statistical software.

### Ethics

According to French law and the French Data Protection Authority, the handling of these data for research purposes was declared to the Data Protection Officer of the Bordeaux university hospital. The study obtained the approval of the Institutional Review Board of the University Hospital of Bordeaux (declaration number CER–BDX-2021-37). Patients (or their relatives, if any) were notified about the anonymized use of their healthcare data via the department's booklet.

## Results

### Flow-chart

**Fig. 1:**
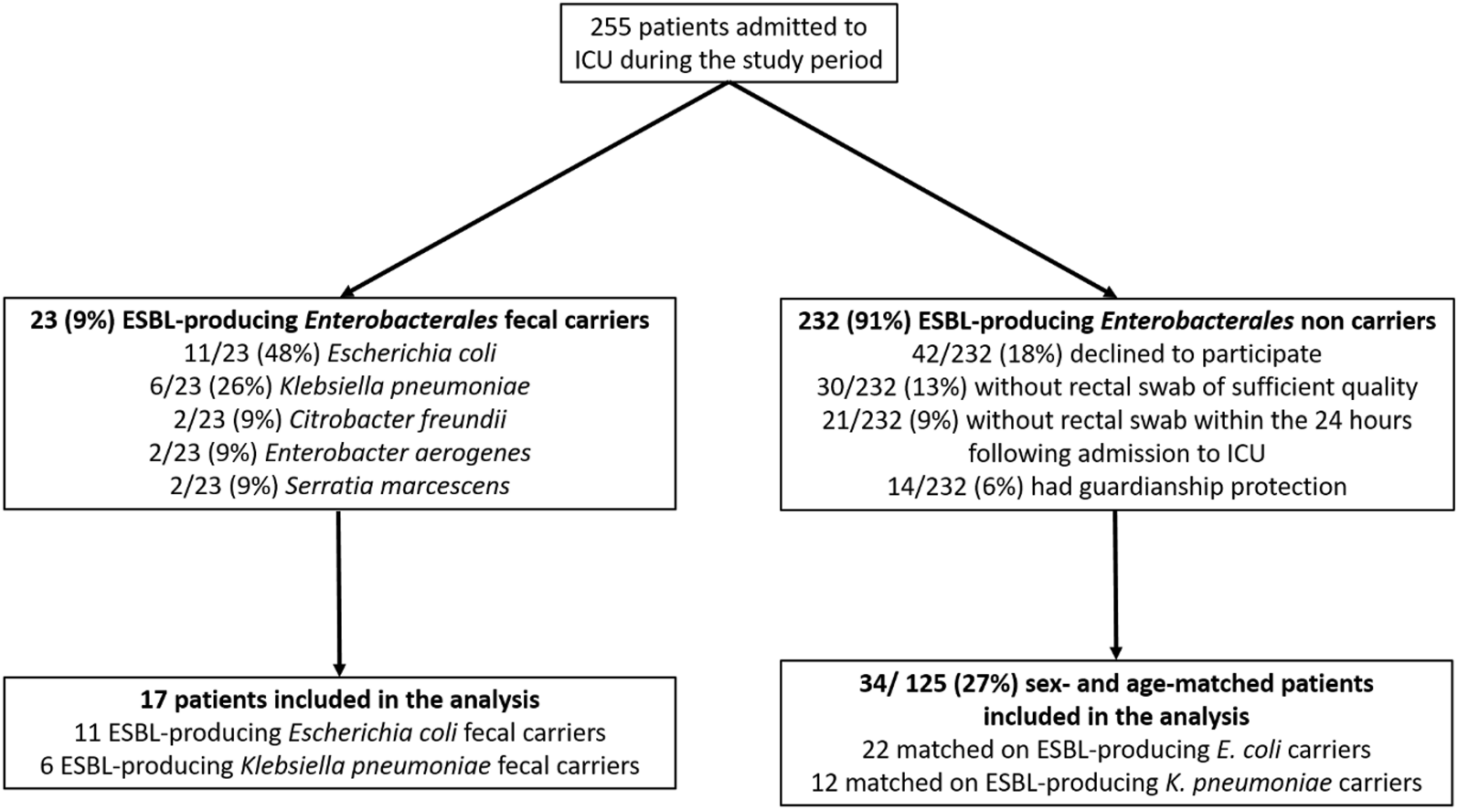
Flow-chart. *ESBL* extended-spectrum beta-lactamase, *ICU* intensive care unit

Among the 255 patients admitted to single centre ICU, 23 (9%) had a faecal carriage with an ESBL-E: 11/23 (48%) *E. coli*, 6/23 (26%) *K. pneumoniae* and 6/23 (8%) *Enterobacter aerogenes, Citrobacter freundii* or *Serratia marcescens* (2 patients for each) (Fig. 1). Because of statistical issues, only ESBL-producing *E. coli* and *K. pneumoniae* faecal carriers were further included in the analysis. In this analysed population, every ESBL-E fecal carriage was community-acquired but 2 ESBL-producing *E. coli* carriage were hospital-acquired prior to admission to ICU.

### ESBL-producing *K. pneumoniae* faecal carriers exhibit a lower α-diversity of gut bacteriobiota, but not mycobiota, than ESBL-producing *E. coli* faecal carriers

**Fig. 2:**
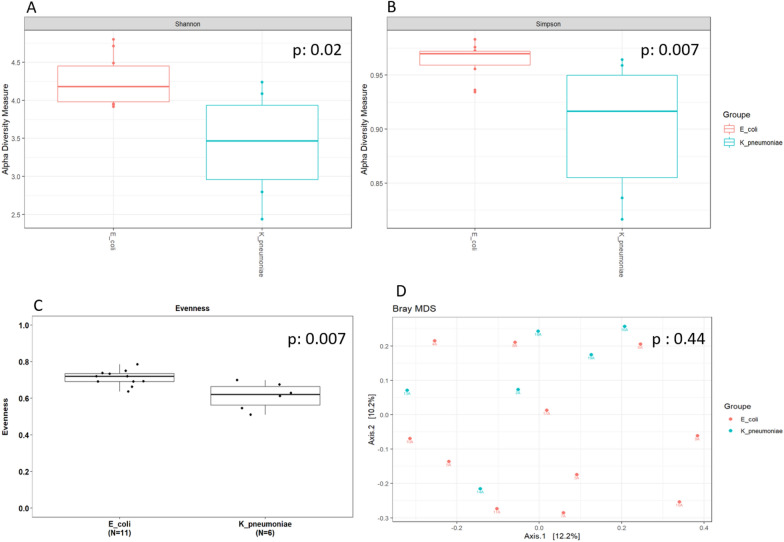
Comparison of gut bacteriobiota between critically ill ESBL-producing *Escherichia coli* and *Klebsiella pneumoniae* faecal carriers. **A**. Boxplot of estimated α-diversity by Shannon index. **B**. Boxplot of estimated α-diversity by Simpson index. **C**. Boxplot of estimated α-diversity by evenness. **D**. Metric Bray–curtis analysis of β-diversity. red: *E. coli*, green: *K. pneumoniae*. Threshold for statistical significance: *p* = 0.05

To fully decipher the impact of bacterial and fungal microbiota on ESBL-E faecal carriage, we first compared clinical characteristics between ESBL-producing *K. pneumoniae* and *E. coli* faecal carriers (Table 1). In brief, ESBL-producing *K. pneumoniae* faecal carriers had higher SAPS2 score (80, IQR: 68–96 vs 55, IQR: 31–68, *p* = 0.007) and a higher rate of acute kidney injury (AKI) (6/6 (100%) *vs* 2/11 (18%), *p* = 0.002) but similar age, sex-ratio, haemodynamic or respiratory failure rates at admission to ICU compared with ESBL-producing *E. coli* faecal carriers. The proportion of patients hospitalized or who received prior antimicrobial therapy within the 3 previous months was not different between ESBL-producing *K. pneumoniae* and *E. coli* faecal carriers nor between each group compared with matched controls (Table 1). Furthermore, the compositions of the gut bacteriobiota and mycobiota were not different between patients who received antimicrobial therapy within the 3 months prior to admission to ICU (Additional file 1: Fig. S3). Gut bacteriobiota α-diversity was lower for ESBL-producing *K. pneumoniae* faecal carriers compared to ESBL-producing *E. coli* faecal carriers regarding Shannon and Simpson indexes, and evenness (*p* = 0.02, *p* = 0.007 and *p* = 0.007, respectively) (Fig. 2A–C), but β-diversity was not different (*p* = 0.44) (Fig. 1D). We did not find any difference in gut mycobiota α- or β-diversity between patients colonized with ESBL-producing *K. pneumoniae* and ESBL-producing *E. coli* (Additional file 1: Fig. S4A–D).

**Table 1:** Patients’ characteristics

Comparison of ESBL-producing *E. coli* and *K. pneumoniae* faecal carriers			
	ESBL-producing *E. coli* (*n* = 11)	ESBL-producing *K. pneumoniae* (*n* = 6)	**p-value**
Characteristics at admission to ICU			
Age (years)	77 [66–84]	76 [57–84]	0.80
Sex (male)	7/11	5/6	0.60
Proton pump inhibitor	2/11	1/6	1.00
Metformin	1/11	0/6	1.00
Hospitalization within the 3 previous months	6/11	5/6	0.33
Median (IQR) length of stay (days)	5 [3–12]	7 [6–12]	0.36
Cause of previous hospitalization	Urinary sepsis: 2 Stroke: 1 Heart failure: 1 Infective endocarditis: 1 Acute hepatitis: 1	Stroke: 1 ACS: 1 Uncompensated diabetes: 1 Diabetic foot ulcer: 1 R-CHOP cures: 1	–
Antimicrobial treatment within the 3 previous months	4/11 Piperacillin/tazobactam: 2 Amoxicilline: 2 Clavulanic acid + amoxicillin: 1	2/6 Piperacillin/tazobactam – linezolide – amikacin: 1 Meropenem – amikacin: 1	0.64
Cause of admission			0.43
Septic shock	4	1	
Hypoxemic ARF	3	1	
Coma	2	2	
Hypercapnic ARF	1	0	
Cardiac arrest	1	1	
Cardiogenic shock	0	1	
SAPS II	55 [31–68]	80 [68–87]	0.007
Septic shock	3/11	2/6	1.00
ARDS	1/11	1/6	1.00
Acute kidney injury	2/11	6/6	0.002
Outcomes			
ICU-mortality	1/11	2/6	0.51

Comparison of ESBL-producing *E. coli* and matched non ESBL-E faecal carriers			
	ESBL-producing *E. coli* (*n* = 11)	matched non ESBL-E (*n* = 22)	*p*-value
Characteristics at admission to ICU			
Age (years)	77 [66–84]	74 [65–78]	0.48
Sex (male)	7/11	14/22	1.00
Proton pump inhibitor	2/11	6/22	0.69
Metformin	1/11	3/22	1.00
Hospitalization within the 3 previous months	6/11	13/22	1.00
Median (IQR) length of stay (days)	5 [3–12]	13 [23–30]	< 0.01
Cause of previous hospitalization	Urinary sepsis: 2 Stroke: 1 Heart failure: 1 Infective endocarditis: 1 Acute hepatitis: 1	Stroke: 3 Pneumonia: 2 ACS: 2 Heart failure: 1 Acute limb ischemia: 1 AECOPD: 1 Chest trauma: 1 Epilepsy: 1 Spine surgery: 1	
Antimicrobial treatment within the 3 previous months	4/11 Piperacillin/tazobactam: 2 Amoxicilline: 2 Clavulanic acid + amoxicillin: 1	9/22 Amoxicilline: 3 Spiramycin: 2 Levofloxacine: 2 3^rd^ generation cephalosporin: 1 Clavulanic acid + amoxicillin: 1	1.00 -
SAPS II	55 [31–68]	70 [45–78]	0.10
Septic shock	3/11	10/22	0.46
ARDS	1/11	2/22	1.00
Acute kidney injury	2/11	14/22	0.03
Outcomes			
ICU-mortality	1/11	5/22	0.64

ESBL-producing *K. pneumoniae* and matched non ESBL-E faecal carriers			
	ESBL-producing *K. pneumoniae*(*n* = 6)	matched non ESBL-E (*n* = 12)	*p*-value
Characteristics at admission to ICU			
Age (years)	76 [57–84]	74 [66–81]	0.89
Sex (male)	5/6	10/12	1.00
Proton pump inhibitor	1/6	5/12	0.60
Metformin	0/6	1/12	1.00
Hospitalization within the 3 previous months	5/6	7/12	0.60
Median (IQR) length of stay (days)	7 [6–12]	12 [5–17]	0.46
Cause of previous hospitalization	Stroke: 1 ACS: 1 Uncompensated diabetes: 1 Diabetic foot ulcer: 1 R-CHOP cures: 1	Stroke: 1 Heart failure: 1 Pneumonia: 1 AECOPD: 1 Epilepsy: 1 Kidney transplantation: 1 Digestive surgery: 1	-
SAPS II	80 [68–87]	65 [54–77]	0.08
Septic shock	2/6	3/12	1.00
ARDS	1/6	4/12	0.61
Acute kidney injury	6/6	7/12	0.11
Antimicrobial treatment during the 3 previous months	2/6 Piperacillin/tazobactam—linezolide—amikacin: 1 Meropenem—amikacin: 1	3/12 Amoxicilline: 2 Clavulanic acid + amoxicilline: 1	0.34 -
Outcomes			
ICU-mortality	2/6	3/12	1.00

Results are presented as proportion for categorical variables and median [interquartile range] for continuous variables

*ACS* acute coronary syndrome, *AECOPD* acute exacerbation of chronic obstructive pulmonary disease, *ARDS* acute respiratory distress syndrome, *ARF* acute respiratory failure, *ICU* intensive care unit, *IQR* interquartile range, *R-CHOP* rituximab-cyclophosphamide-doxorubicin-vincristine-prednisone, *SAPS* simplified acute physiology score II

Threshold for statistical significance: *p* = 0.05.

As it seems of crucial importance to consider not only the phenotype of antimicrobial resistance but also the bacterial species carrying the antimicrobial resistance determinant(s), we further compare independently the microbiota composition of ESBL-producing *K. pneumoniae* and *E. coli* faecal carriers to sex- and age-matched non ESBL-E faecal carriers in 1:2 proportion.

### Gut bacteriobiota and mycobiota diversities are not different between ESBL-producing *E. coli* faecal carriers and non-carriers but microbial community enrichment was associated with the absence of ESBL-E faecal carriage

**Fig. 3:**
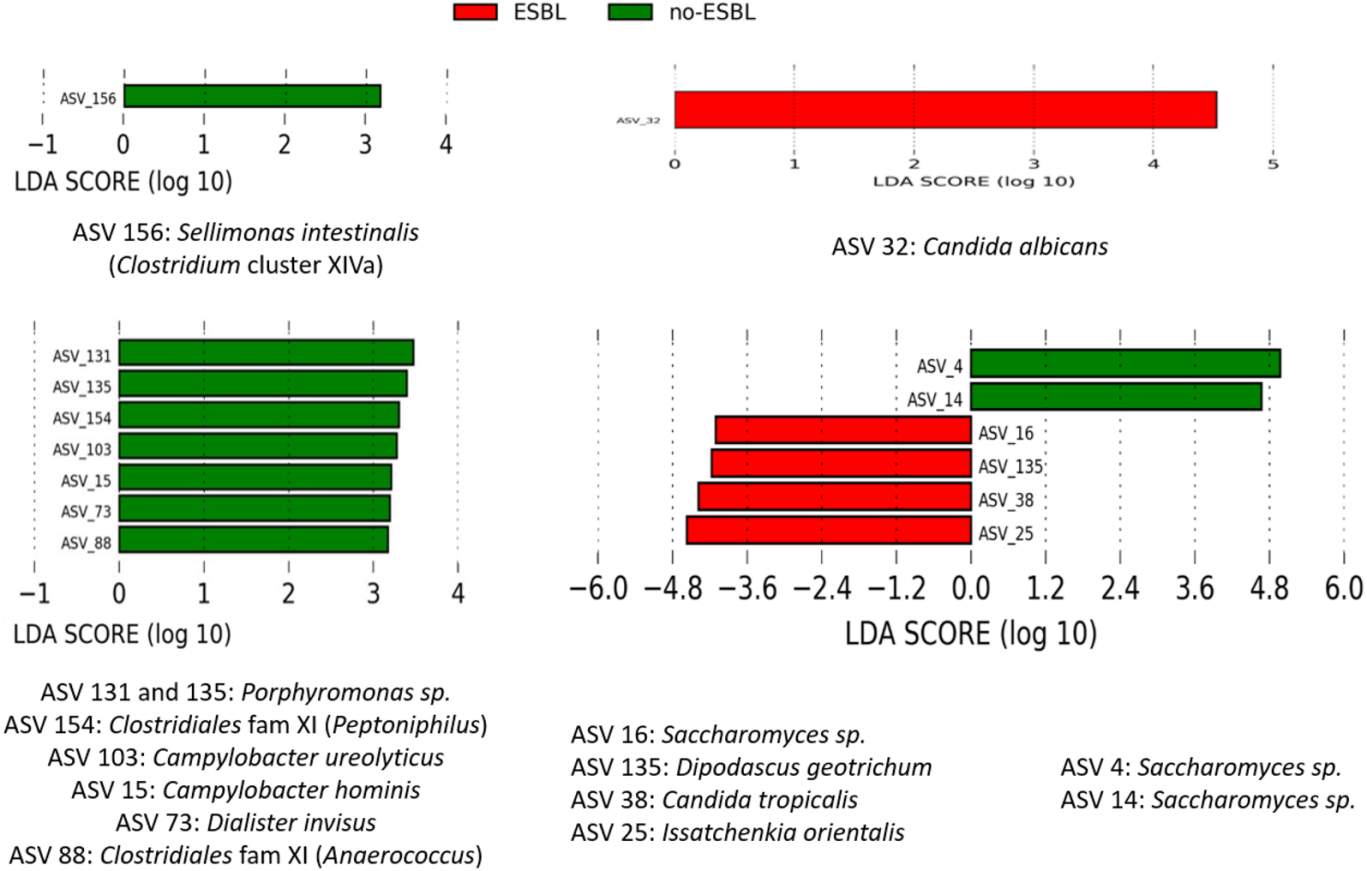
Comparison of bacterial and fungal species abundances between critically ill ESBL-E fecal carriers and matched non ESBL-E faecal carriers. **A**. Bacterial species abundances between ESBL-producing *E. coli* fecal carriers and matched non ESBL-E faecal carriers. **B**. Fungal species abundances between ESBL-producing *E. coli* fecal carriers and matched non ESBL-E faecal carriers. **C**. Bacterial species abundances between ESBL-producing *K. pneumoniae* fecal carriers and matched non ESBL-E faecal carriers. **D**. Fungal species abundances between ESBL-producing *K. pneumoniae* fecal carriers and matched non ESBL-E faecal carriers LefSe analysis with linear discriminant analysis (LDA). Threshold for statistical significance: LDA > 3log. ASV: amplicon sequence variant

ESBL-producing *E. coli* faecal carriers’ characteristics were similar to non ESBL-E faecal carriers excepting a lower rate of AKI (*p* = 0.03) (Table 1). Both α and β diversities of gut bacteriobiota and mycobiota were not dissimilar between ESBL-producing *E. coli* faecal carriers and non ESBL-E faecal carriers (Additional file 1: Figs. S5, S6A–D). However, using LDA method to compare gut microbiota composition between ESBL-producing *E. coli* faecal carriers and non ESBL-E faecal carriers, the presence of *Sellimonas intestinalis* was associated with the absence of ESBL-producing *E. coli* faecal carriage (LDA score > 3) (Fig. 3A). To the contrary, the presence of *Candida albicans* appeared to be associated with ESBL-producing *E. coli* faecal carriage (Fig. 3B).

### ESBL-producing *K. pneumoniae* faecal carriers exhibit a lower α-diversity of gut bacteriobiota but not mycobiota than non-carriers

ESBL-producing *K. pneumoniae* faecal carriers had higher SAPSII scores and AKI rates compared to non ESBL-E faecal carriers despite those differences being non-significant (p = 0.08 and p = 0.11, respectively) (Table 1).

**Fig. 4:**
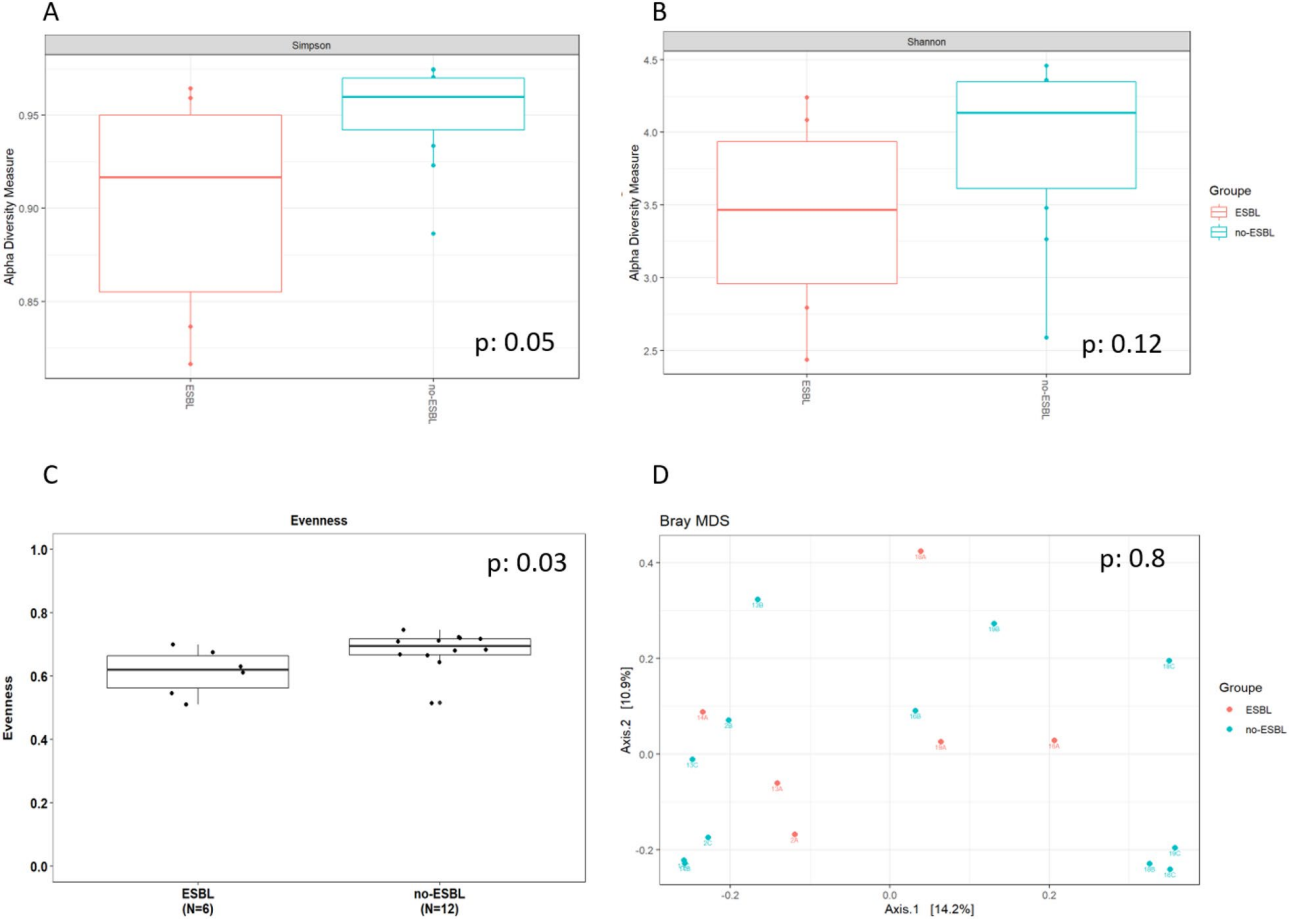
Comparison of gut bacteriobiota between critically ill ESBL-producing *Klebsiella pneumoniae* faecal carriers (in red) and matched non ESBL-E faecal carriers (in blue). **A**. Boxplot of estimated α-diversity by Shannon index. **B**. Boxplot of estimated α-diversity by Simpson index. **C**. Boxplot of estimated α-diversity by evenness. **D**. Metric Bray–curtis analysis of β-diversity. Threshold for statistical significance: *p* = 0.05

Gut bacteriobiota α-diversity was lower in ESBL-producing *K. pneumoniae* faecal carriers compared to non ESBL-E faecal carriers (respectively for Shannon and Simpson indexes and evenness p = 0.12, p = 0.05 and p = 0.03, respectively) (Fig. 4A–C), but β-diversity was not different (p = 0.8) (Fig. 4D). We did not find any difference in gut mycobiota α- or β-diversity (Additional file 1: Fig. S7A–D).

### Identification of probiotic candidates for ESBL-producing *K. pneumoniae* tailored decontamination

Using LDA analysis to detect significant microbial community enrichment, we identified a consortium of bacteria associated with the absence of ESBL-producing *K. pneumoniae* faecal carriage (LDA score > 3) including *Campylobacter hominis*, *Campylobacter ureolyticus*, *Porphyromonas sp., Clostridiales fam XI* (*Peptoniphilus* and *Anaerococcus*) (Fig. 3C).

Analyzing fungal species, amplicon sequence variants (ASV) related to *Issatchenkia orientalis* (*Candida krusei*), *Dipodascus geotrichum* (*Geotrichum candidum*) and to *Candida tropicalis* were significantly associated with ESBL-producing *K. pneumoniae* faecal carriage; while ASV related to *Saccharomyces* species were associated either with the colonization or the absence of colonization with ESBL-producing *K. pneumoniae* (Fig. 3D).

## Discussion

To the best of our knowledge, this study is the first to compare the gut microbiota compositions between ESBL-producing *K. pneumoniae* and *E. coli* carriers and to include the analysis of gut mycobiota. Notably, we identified a decreased α-diversity of gut bacteriobiota in ESBL-producing *K. pneumoniae* compared to ESBL-producing *E. coli* faecal carriers. Gut microbiota diversities were not different between ESBL-E producing *E. coli* faecal carriers and non ESBL-E faecal carriers, but ESBL-producing *K. pneumoniae* faecal carriers exhibited a lower gut bacteriobiota α-diversity, compared to non ESBL-E faecal carriers.

Two previous study investigated the link between gut bacteriobiota composition and gut colonization with MDRO in ICU, one finding no difference in gut bacteriobiota α- and β-diversities between 13 MDRO carriers and 18 non-carriers [14] and the other that Family XI and *Prevotellaceae* families are more abundant in non-carriers than in MDRO carriers [15]. Nevertheless, these studies included ESBL, carbapenemase and/or high-level AmpC producing *Enterobacterales*, ceftazidime-resistant *Pseudomonas aeruginosa* and *Stenotrophomonas maltophilia.* The mixing of different bacterial species with different pathogenicity abilities, virulence factors and diverse interactions with other microbial species may provide heterogeneity in the analysis.

In our study, gut microbiota α-diversity was found to be different between ESBL *K. pneumoniae* fecal carriers and other groups, but not β diversity. This apparent discrepancy is not unusual in microbiota studies as α-diversity reflects the within sample diversity taking into account the number of different species and their relatives abundances in this sample whereas the β-diversity reflects between sample diversity and how similar is the composition of the microbiota between the samples included in the analysis.

The decreased α-diversity of gut bacteriobiota in patients colonized with ESBL-producing *K. pneumoniae* compared with ESBL-producing *E. coli* faecal carriers and non-carriers of ESBL-producing *Enterobacterales*, could be, at least partially, explained by the fact that these patients are frailer with more comorbidities as previously described [13] and that increased host frailty is associated with altered gut bacteriobiota composition [16]. This enhances the importance to take the bacterial specie and not only the antimicrobial resistance mechanism into account when assessing the impact of gut microbiota on the fecal carriage of MDRO. Moreover, the correlation between the decrease in α-diversity of the gut microbiota and the inoculum size of the colonizing ESBL-E will soon be feasible thanks to the recently improved sequencing accuracy of the long-read sequencing technologies.

Regarding the community and long-term facility residents, a first study did not find any association between gut microbiota dynamics and colonization with extended-spectrum cephalosporin-resistant *Enterobacteriaceae* in healthy Swiss travellers to India [17]. Another study did not find either any difference in gut microbiota α-diversity between ESBL-E carriers compared with non-carriers in Thaïland community but find a significant dissimilarity (β-diversity) between the 2 groups, probably reflecting the fact that ESBL-E faecal carriage was significantly lower in farmers than in people with other professions. The presence of *Bacteroides uniformis* was statistically more abundant in ESBL-E non-carriers than in carriers [18]. The same comparison in the Amerindian Wayampi community showed that ESBL-E faecal carriers had a lower gut bacteriobiota α-diversity than non-carriers while taxa belonging to the genera *Desulfovibrio* and *Oscillospira* (Ruminococcaceae) were associated with the absence of ESBL-E faecal carriage and *Prevotella* was associated with ESBL-E faecal carriage [19]. Comparison of gut bacteriobiota between ESBL-E faecal carriers and non-carriers, not in the community but in nursing home residents, found a decreased α-diversity in ESBL-E faecal carriers with a depletion in butyrate-producing bacterial species and an enrichment in succinate-producing bacterial species [20]. While the impact of microbiota on MDRO is mediated by indirect mechanisms including local and systemic immune activation and by direct mechanisms including competition for nutrients between species composing the microbiota and direct toxicity, none of these studies took ESBL-E bacterial species into account. Only one study specifically assessed the impact of ESBL-E bacterial species in a mice model of ESBL-producing *K. pneumoniae* gut colonization and concluded that the microbial composition plays a primary role in MDR-colonization rate, whereas the antibiotic susceptibility of individual MDR strains affects this process in a lesser extent [21] enhancing the relevance of taking into account the bacterial specie carrying the resistance mechanism.

Regarding the identification of potential probiotics for tailored ESBL-producing *E. coli* faecal carriage decontamination, *S. intestinalis* could be an interesting candidate as it belongs to *Clostridiales* cluster XIVa which includes several butyrate-producing or bacteriocin-producing bacterial species. *S. intestinalis* has only been discovered in 2016 and requires further investigation [22–24]. The fact that *C. albicans* presence is associated with ESBL-producing *E. coli* faecal carriage is of major interest as *C. albicans* wall components provide a higher level of *E. coli* resistance to ofloxacin within a polymicrobial biofilm [25]. Furthermore, mannose and mannans composing *C. albicans* wall seem to inhibit *E. coli* phagocytosis by macrophages [26].

Regarding the identification of potential probiotics for tailored ESBL-producing *K. pneumoniae* faecal carriage decontamination, few data exist about the interactions between identified bacterial and fungal species and *K. pneumoniae*. Several studies suggest that bacterial species belonging to *Campylobacter* genus are associated with a decreased inoculum of *K. pneumoniae*. In fact, broilers fed with *Campylobacter jejuni* have lower amount of *K. pneumoniae* isolated at both ileal and caecal areas [27]. This antagonism seems to be two-sided as *K. pneumoniae* is also able to products metabolites that decrease *C. jejuni* growth [28, 29]. In this study, we identified *Saccharomyces sp.* to be associated both with ESBL-producing *K. pneumoniae* carriage and non-carriage. Explaining this apparent discrepancy, species belonging to *Saccharomyce*s genus have been reported to have contrary interactions with *K. pneumoniae*. One one hand, *Saccharomyces boulardii* supernatant inhibits *K. pneumoniae* metabolism and growth [30]. On the other hand, *Saccharomyces cerevisiae* seems to stimulate *K. pneumoniae* growth via the production of 3-hydroxypropionic acid and quinone pyrroloquinolin [31, 32]. Regarding *Candida tropicalis* and *Issatchenkia orientalis* (also known as *Candida krusei*), only one study assessed their potential interaction with *K. pneumoniae* and did not find any effect of *K. pneumoniae* lipopolysaccharides on in vitro biofilm formation of these *Candida* species [33] and no data are available for *Dipodascus geotrichum*.

The potential role of gut mycobiota should not be underestimated in the resistance to colonization with ESBL-E as demonstrated by the identification of fungal species associated with the presence or absence of colonizing ESBL-E. Besides the direct inter-kingdoms interactions occurring in the microbiota [34], commensal fungi also modulate local and systemic immunity [35].

The main limitation is the monocentric character of our study and the relative small number of patients. Age- and sex-matching was performed to limit microbiota composition variability as these 2 factors are key determinants of microbiota composition [36–38]. Patients were not matched on comorbidities and severity as ESBL-producing *K. pneumoniae* fecal carriers have more cardiovascular and neurological comorbidities and are more immunosuppressed than ESBL-producing *E. coli* fecal carriers and ESBL-E non carriers [12, 13]. The absence of matching on these parameters would have been a bias if we were looking for an independent association between the gut microbiota composition and the ESBL-E colonization or if we wanted to explain the causes of these alterations. To the contrary, we aimed to compare these gut microbiota alterations between ESBL-E fecal carriers and non-carriers, whether or not these alterations are due to the comorbidities of the patients or the severity of the disease. Besides, the use of DADA2 pipeline will allow comparison of the results from different teams if the same primers are used for gene amplification, thus allowing reproducibility assessment [9, 39]. Interestingly, we did not find significant difference in the proportion of patients hospitalized within the 3 previous months which could be consistent with the global dissemination of E-ESBL in the community. Nevertheless, as discussed above, ESBL-producing *K. pneumoniae* fecal carriers had higher SAPS2 score and a higher rate of AKI compared to ESBL-producing *E. coli* fecal carriers and a trend compared to non fecal-carriers which could partly explain our results. In fact, it is highly plausible that critical illness [40] and frailty [16] could reduce gut microbial diversity thus impairing host colonization resistance to pathogens [4]. This has been suggested long before the onset of metagenomics tools to investigate gut microbiota composition as aged, frail patients have been demonstrated to have high proportion of oropharyngeal colonization with Gram-negative bacilli even in the absence of previous antimicrobial therapy [41]. If so, persistence of the underlying condition responsible for this frailty or persistence of the critical illness could impair the long-term efficacy of exogenic gut microbiota diversity restoration for providing colonization resistance and could lead to treatment failure or need for repeated probiotics administration [5]. Another limitation is the lack of causality demonstration. To get beyond the association links provided in this study, animal models are needed to assess the real ability of identified probiotic candidates to eradicate ESBL-E faecal carriage.

Moreover, concerns exist about the translocation of probiotics given to patients with increased gut permeability [42] as occurring in ICU patients. In vitro studies are thus needed to identify the underlying mechanisms of micro-organisms inhibition (i.e., bacteriocin production, environment pH modulation via butyrate production, metabolic antagonism, …) as tailored decontamination could also be mediated via microbial metabolites and/or products.

## Conclusions

The composition of the gut microbiota differs between ESBL-producing *E. coli* and ESBL-producing *K. pneumoniae* faecal carriers: ESBL-producing *K. pneumoniae* faecal carriers exhibiting lower gut bacteriobiota α-diversity compared to ESBL-producing *E. coli* faecal carriers and ESBL-E non carriers. These data suggest that bacterial species, in addition to the mechanism of antimicrobial resistance, should be taken into account when investigating the role of gut microbiota in the resistance to gut colonization with ESBL-E. This approach could lead to identification of probiotic candidates for tailored gut decontamination in ESBL-E faecal carriers admitted to ICU.

## Supplementary Information

**Additional file 1: Figure S1.** Non metric Bray-curtis analysis of β-diversity of the V3-V4 sequencing run. XXA, B, and C samples: gut bacteriobiota samples. GXX: lung bacteriobiota samples. BlancV3-V4: negative control. Mock: mock community. **Figure S2.** Non metric Bray-curtis analysis of β-diversity of ITS2 sequencing run. XXA, B, and C samples: gut mycobiota samples. GXX: lung mycobiota samples. Blanc1 and blanc2: negative controls. Mock: mock community. **Figure S3.** Comparison of gut bacteriobiota and mycobiota between critically ill ESBL-producing Enterobacteriales fecal carriers and non-carriers. A. Boxplot of estimated α-diversity for gut bacteriobiota by Shannon index. B. Boxplot of estimated α-diversity for gut bacteriobiota by Simpson index. C. Metric Bray-curtis analysis of β-diversity for gut bacteriobiota. Threshold for statistical significance: p=0.05. D. Boxplot of estimated α-diversity for gut mycobiota by Shannon index. E. Boxplot of estimated α-diversity for gut mycobiota by Simpson index. F. Metric Bray-curtis analysis of β-diversity for gut mycobiota. Threshold for statistical significance: p=0.05. ATB: prior antimicrobial therapy within the 3 previous months. no-ATB: no prior antimicrobial therapy within the 3 previous months. **Figure S4.** Comparison of gut mycobiota between critically ill ESBL-producing Escherichia coli and Klebsiella pneumoniae faecal carriers. A. Boxplot of estimated α-diversity by Shannon index. B. Boxplot of estimated α-diversity by Simpson index. C. Boxplot of estimated α-diversity by evenness. D. Metric Bray-curtis analysis of β-diversity. red: E. coli, green: K. pneumoniae. Threshold for statistical significance: p=0.05. **Figure S5.** Comparison of gut bacteriobiota between critically ill ESBL-producing Escherichia coli (in red) and matched non ESBL-E (in blue) faecal carriers. A. Boxplot of estimated α-diversity by Shannon index. B. Boxplot of estimated α-diversity by Simpson index. C. Boxplot of estimated α-diversity by evenness. D. Metric Bray-curtis analysis of β-diversity. Threshold for statistical significance: p=0.05. **Figure S6.** Comparison of gut mycobiota between critically ill ESBL-producing Escherichia coli faecal carriers (in red) and matched non ESBL-E faecal carriers (in blue). A. Boxplot of estimated α-diversity by Shannon index. B. Boxplot of estimated α-diversity by Simpson index. C. Boxplot of estimated α-diversity by evenness. D. Metric Bray-curtis analysis of β-diversity. Threshold for statistical significance: p=0.05. **Figure S7.** Comparison of gut mycobiota between critically ill ESBL-producing Klebsiella pneumoniae faecal carriers (in red) and matched non ESBL-E faecal carriers (in blue). A. Boxplot of estimated α-diversity by Shannon index. B. Boxplot of estimated α-diversity by Simpson index. C. Boxplot of estimated α-diversity by evenness. D. Metric Bray-curtis analysis of β-diversity. Threshold for statistical significance: p=0.05.

## Data Availability

The 16S rRNA gene and ITS2 sequences have been submitted to the European Nucleotide Archive (Accession N◦ ERP134947). The scripts used for bioinformatics analysis during the current study are available from the corresponding author on reasonable request.

## Abbreviations

AKI: Acute kidney injury
ASV: Amplicon sequence variant
ESBL-E: Extended spectrum beta-lactamase producing *Enterobacteriales*
ICU: Intensive care unit
IQR: Interquartile range
LDA: Linear discriminant analysis
LefSe: LDA effect size
MDRO: Multi-drug resistant organism
PERMANOVA: Permutational multivariate ANOVA
SAPSII: Simplified acute physiology score II
VRE: Vancomycin-resistant *Enterococcus faecium*
