# Supplementary material for: Bridging gut microbiota composition with extended-spectrum beta-lactamase Enterobacteriales faecal carriage in critically ill patients (microbe cohort study)
Source: Ann Intensive Care. 2023 Apr 4;13:25. doi: 10.1186/s13613-023-01121-0 (PMC10073396; doi:10.1186/s13613-023-01121-0)
Supplement: Supplementary file 1 — Additional file 1: Figure S1. Non metric Bray-curtis analysis of β-diversity of the V3-V4 sequencing run. XXA, B, and C samples: gut bacteriobiota samples. GXX: lung bacteriobiota samples. BlancV3-V4: negative control. Mock: mock community. Figure S2. Non metric Bray-curtis analysis of β-diversity of ITS2 sequencing run. XXA, B, and C samples: gut mycobiota samples. GXX: lung mycobiota samples. Blanc1 and blanc2: negative controls. Mock: mock community. Figure S3. Comparison of gut bacteriobiota and mycobiota between critically ill ESBL-producing Enterobacteriales fecal carriers and non-carriers. A. Boxplot of estimated α-diversity for gut bacteriobiota by Shannon index. B. Boxplot of estimated α-diversity for gut bacteriobiota by Simpson index. C. Metric Bray-curtis analysis of β-diversity for gut bacteriobiota. Threshold for statistical significance: p=0.05. D. Boxplot of estimated α-diversity for gut mycobiota by Shannon index. E. Boxplot of estimated α-diversity for gut mycobiota by Simpson index. F. Metric Bray-curtis analysis of β-diversity for gut mycobiota. Threshold for statistical significance: p=0.05. ATB: prior antimicrobial therapy within the 3 previous months. no-ATB: no prior antimicrobial therapy within the 3 previous months. Figure S4. Comparison of gut mycobiota between critically ill ESBL-producing Escherichia coli and Klebsiella pneumoniae faecal carriers. A. Boxplot of estimated α-diversity by Shannon index. B. Boxplot of estimated α-diversity by Simpson index. C. Boxplot of estimated α-diversity by evenness. D. Metric Bray-curtis analysis of β-diversity. red: E. coli, green: K. pneumoniae. Threshold for statistical significance: p=0.05. Figure S5. Comparison of gut bacteriobiota between critically ill ESBL-producing Escherichia coli (in red) and matched non ESBL-E (in blue) faecal carriers. A. Boxplot of estimated α-diversity by Shannon index. B. Boxplot of estimated α-diversity by Simpson index. C. Boxplot of estimated α-diversity by evenness. [file 13613_2023_1121_MOESM1_ESM.docx]

**Definition of comorbidities**

Comorbidities were defined as follows: chronic obstructive pulmonary disease and asthma were defined according to lung function testing. Chronic heart failure was defined according to transthoracic echocardiography and chronic coronary disease based on stress test or percutaneous coronary intervention. Other comorbidities included history of chronic kidney disease, immunosuppression (drugs, haematological disease, blood marrow transplantation, solid organ transplantation, plasma exchanges indicated by autoimmune disorders, human immunodeficiency virus infection), simplified acute physiology score II (SAPSII). Acute respiratory distress syndrome was defined according to Berlin’s criteria [8], septic shock according to SEPSIS-III definition [9] and acute kidney injury (AKI) to KDIGO guidelines [10].

**Additional file Figure S1** Non metric Bray-curtis analysis of β-diversity of the V3-V4 sequencing run. XXA, B, and C samples: gut bacteriobiota samples. GXX: lung bacteriobiota samples. BlancV3-V4: negative control. Mock: mock community.


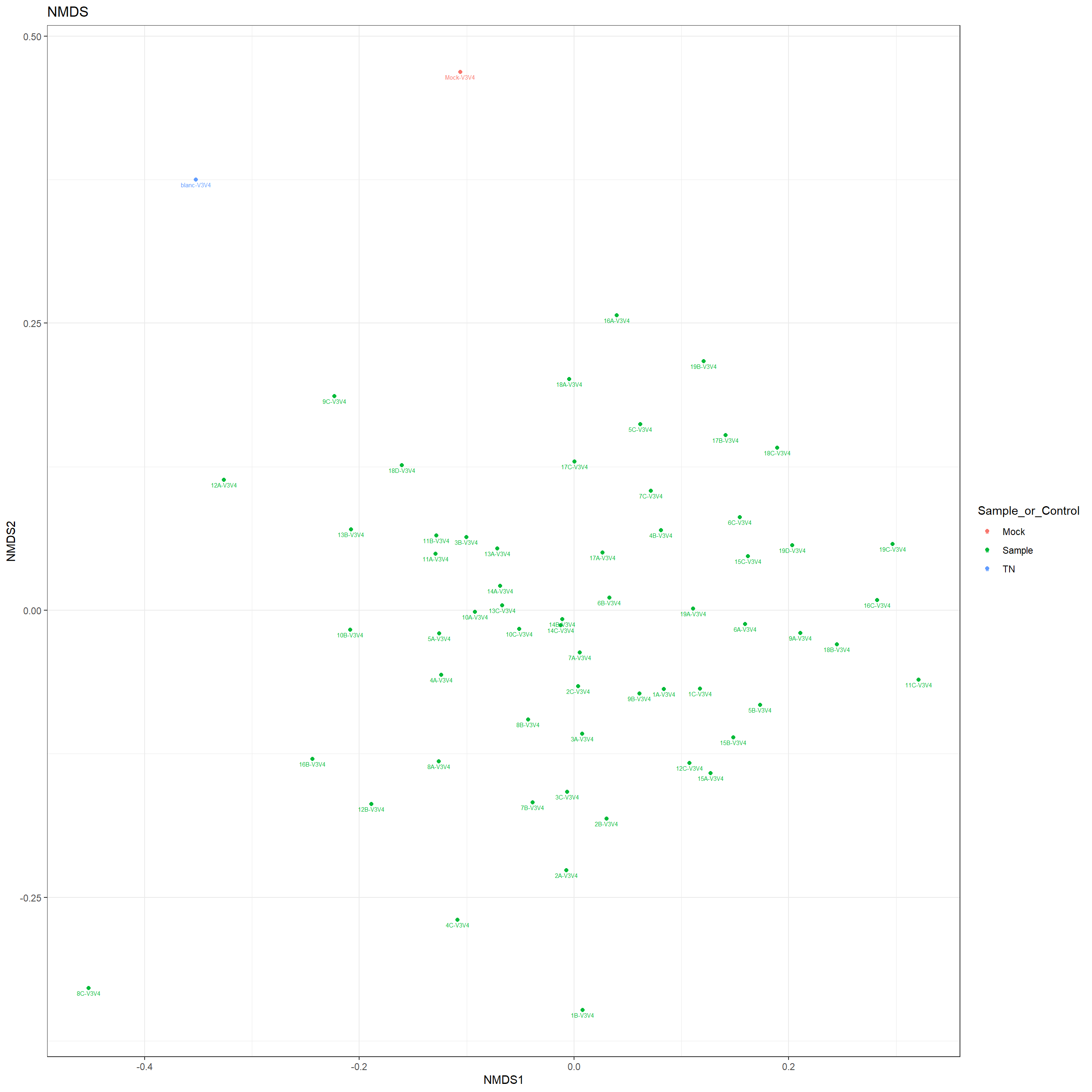


**Additional file Figure S2** Non metric Bray-curtis analysis of β-diversity of ITS2 sequencing run. XXA, B, and C samples: gut mycobiota samples. GXX: lung mycobiota samples. Blanc1 and blanc2: negative controls. Mock: mock community.


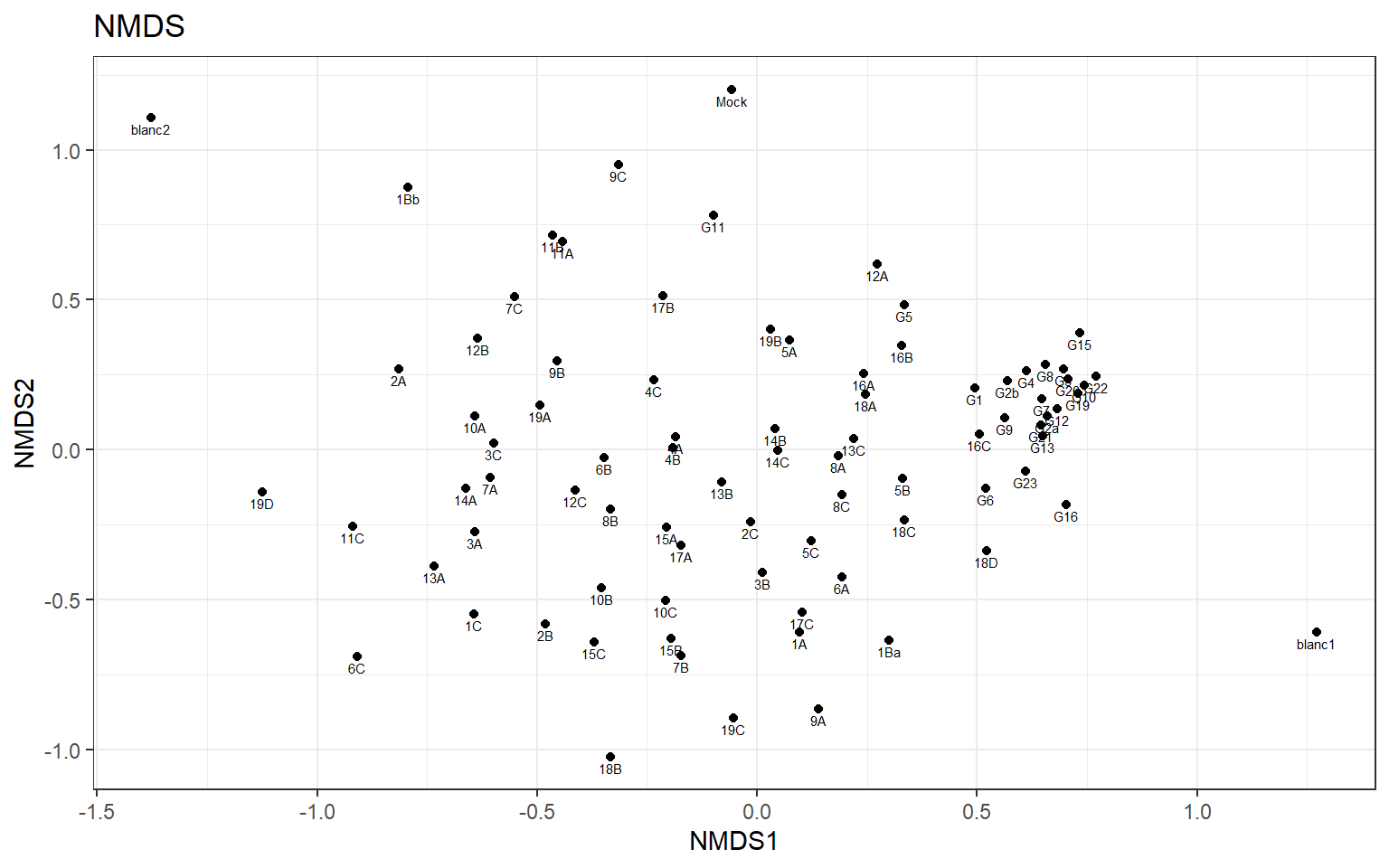


**Additional file Figure S3 Comparison of gut bacteriobiota and mycobiota between critically ill ESBL-producing Enterobacteriales fecal carriers and non-carriers.** A. Boxplot of estimated α-diversity for gut bacteriobiota by Shannon index. B. Boxplot of estimated α-diversity for gut bacteriobiota by Simpson index. C. Metric Bray-curtis analysis of β-diversity for gut bacteriobiota. Threshold for statistical significance: p=0.05. D. Boxplot of estimated α-diversity for gut mycobiota by Shannon index. E. Boxplot of estimated α-diversity for gut mycobiota by Simpson index. F. Metric Bray-curtis analysis of β-diversity for gut mycobiota. Threshold for statistical significance: p=0.05. ATB: prior antimicrobial therapy within the 3 previous months. no-ATB: no prior antimicrobial therapy within the 3 previous months.

**
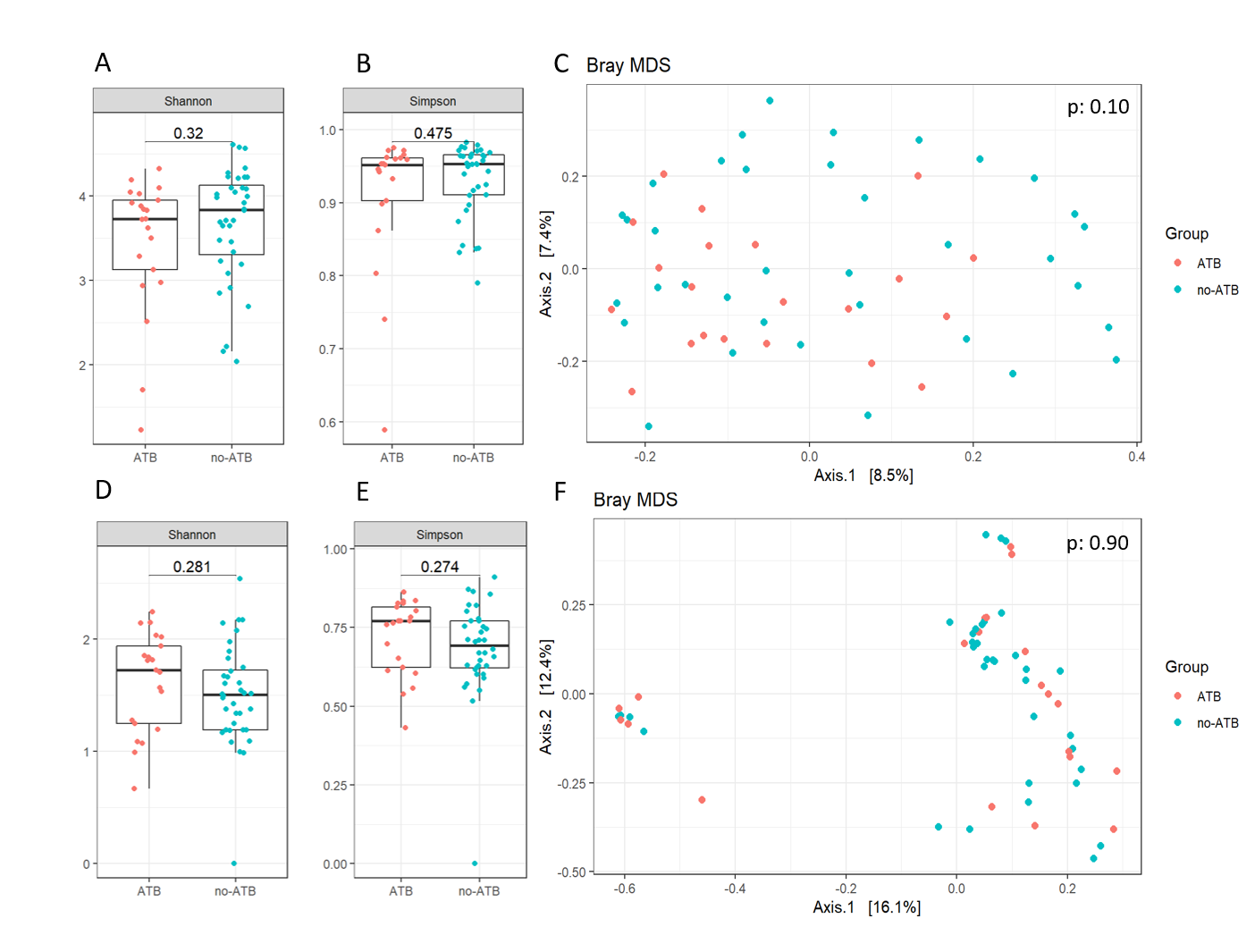
**

**Additional file Figure S4 Comparison of gut mycobiota between critically ill ESBL-producing *Escherichia coli* and *Klebsiella pneumoniae* faecal carriers.** A. Boxplot of estimated α-diversity by Shannon index. B. Boxplot of estimated α-diversity by Simpson index. C. Boxplot of estimated α-diversity by evenness. D. Metric Bray-curtis analysis of β-diversity. red: *E. coli*, green: *K. pneumoniae*. Threshold for statistical significance: p=0.05.


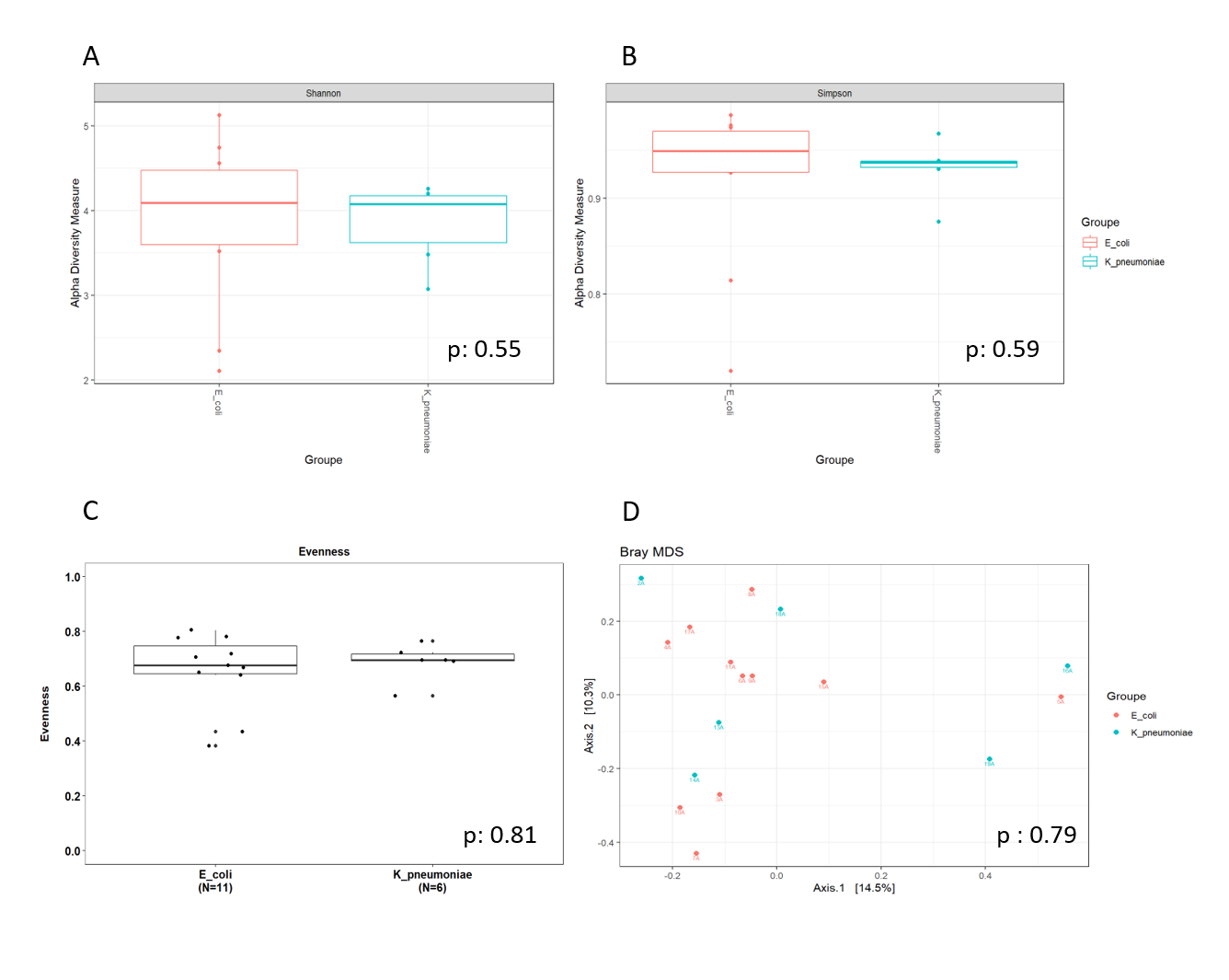


**Additional file Figure 5 Comparison of gut bacteriobiota between critically ill ESBL-producing *Escherichia coli* (in red) and matched non ESBL-E (in blue) faecal carriers.** A. Boxplot of estimated α-diversity by Shannon index. B. Boxplot of estimated α-diversity by Simpson index. C. Boxplot of estimated α-diversity by evenness. D. Metric Bray-curtis analysis of β-diversity. Threshold for statistical significance: p=0.05.


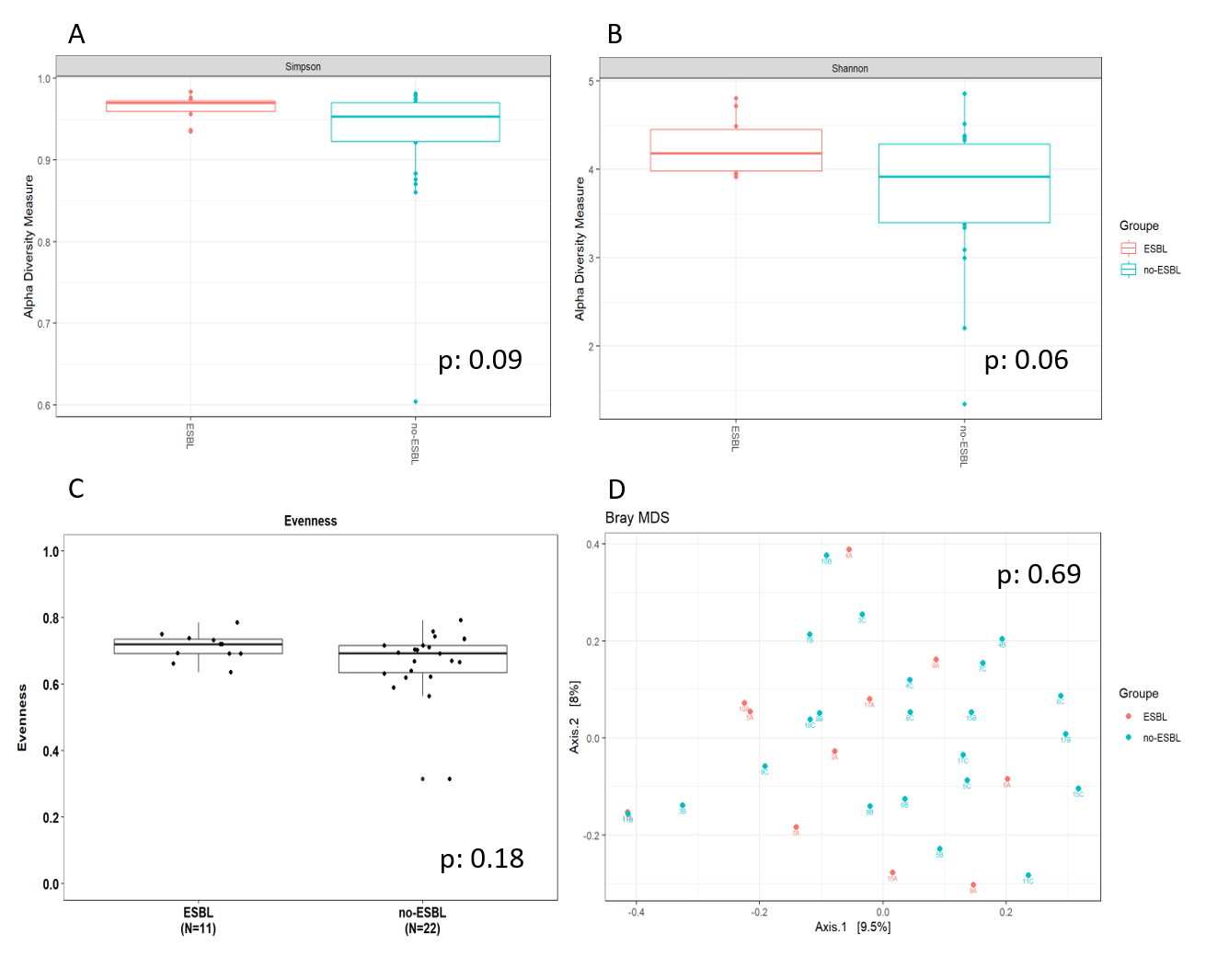


**Additional file Figure S6 Comparison of gut mycobiota between critically ill ESBL-producing *Escherichia coli* faecal carriers (in red) and matched non ESBL-E faecal carriers (in blue).** A. Boxplot of estimated α-diversity by Shannon index. B. Boxplot of estimated α-diversity by Simpson index. C. Boxplot of estimated α-diversity by evenness. D. Metric Bray-curtis analysis of β-diversity. Threshold for statistical significance: p=0.05.


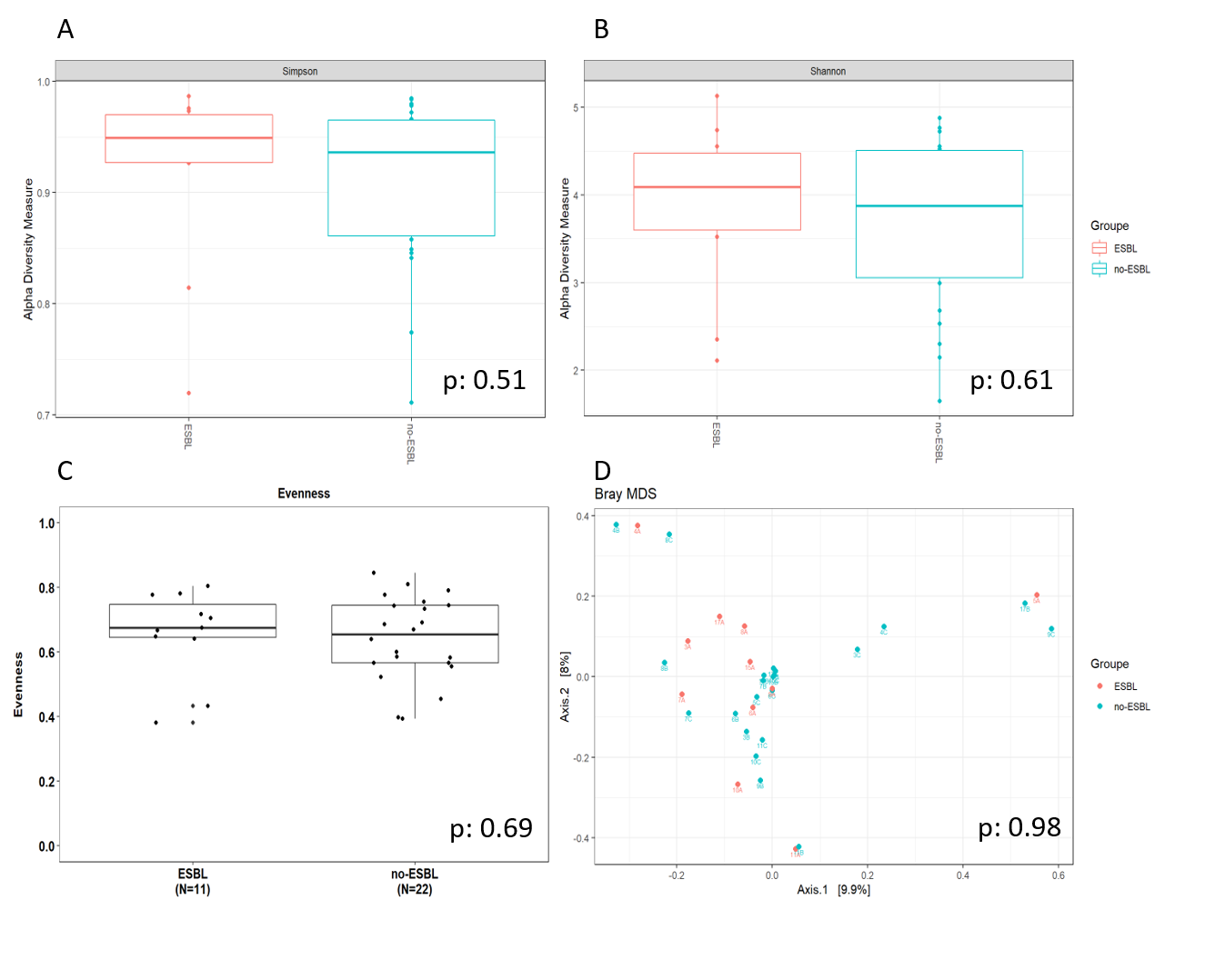


**Additional file Figure S7 Comparison of gut mycobiota between critically ill ESBL-producing *Klebsiella pneumoniae* faecal carriers (in red) and matched non ESBL-E faecal carriers (in blue).** A. Boxplot of estimated α-diversity by Shannon index. B. Boxplot of estimated α-diversity by Simpson index. C. Boxplot of estimated α-diversity by evenness. D. Metric Bray-curtis analysis of β-diversity. Threshold for statistical significance: p=0.05.


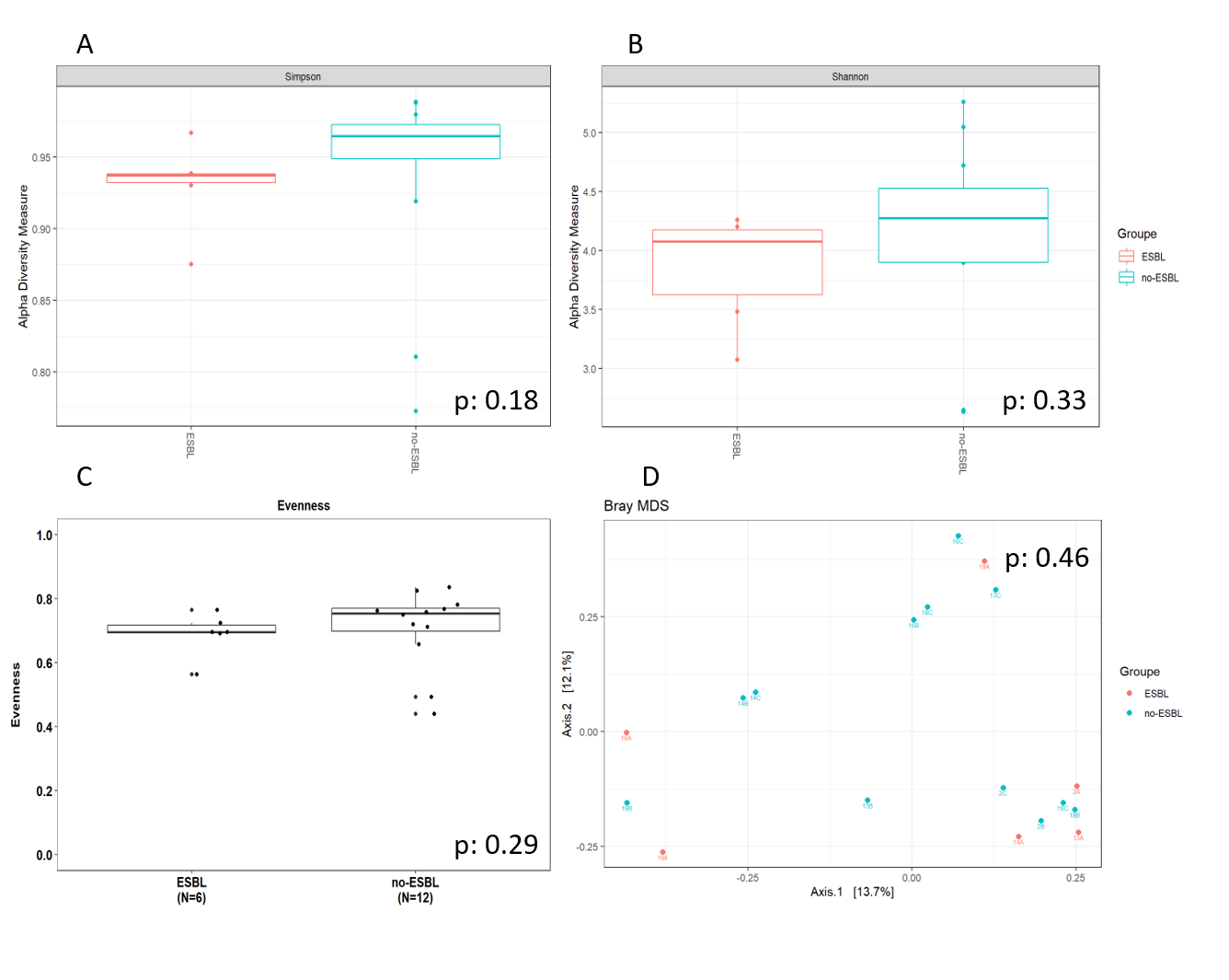


**Mock**

**ZymoBIOMICS® Microbial Community Standard**

*Listeria monocytogenes* - 12%

*Pseudomonas aeruginosa* - 12%

*Bacillus subtilis* - 12%

*Escherichia coli* - 12%

*Salmonella enterica* - 12%

*Lactobacillus fermentum* - 12%

*Enterococcus faecalis* - 12%

*Staphylococcus aureus* - 12%

*Saccharomyces cerevisiae* - 2%

*Cryptococcus neoformans* - 2%

**r16SRNA coding gene home-made mock microbial community standards**

*Streptococcus mitis* (400 µL)

*Streptococcus oralis* (400 µL)

*Pseudomonas aeruginosa* (200 µL)

*Stenotrophomonas maltophilia* (200 µL)

*Staphylococcus epidermidis* (400 µL)

*Staphylococcus aureus* (400 µL)

*Acinetobacter baumannii* (400 µL)

*Klebsiella pneumoniae* (200 µL)

*Proteus mirabilis* (200 µL)

*Serratia marcescens* (400 µL)

*Lactobacillus spp.* (200 µL)

*Escherichia coli* (ATCC 25922) (200 µL)

*Enterobacter cloacae* (200µL)

*Enterococcus faecalis* (ATCC 29212) (400 µL)

**ITS2 coding gene home-made mock microbial community standards**

*Scedosporium apiospermum* (*Pseudallescheria boydii*) (200 µL)

*Mucor circillenoides* (400 µL)

*Candida lusitaniae* (*Clavispora lusitaniae*) (400 µL)

*Scedosporium aurantiacum* (400 µL)

*Lomentospora prolificans* (*Scedosporium prolificans*) (200 µL)

*Aspergillus fumigatus* (400 µL)

*Aspergillus flavus* (200 µL)

*Aspergillus terreus* (200 µL)

*Penicillium griseofulvum* (200 µL)

*Fusarium solani* (200 µL)

*Candida dubliniensis* (400 µL)

*Candida albicans* (ATCC 5314) (400 µL)

*Rhodotorula mucilaginosa* (400 µL)

*Exophiala dermatitidis* (400 µL)

**Script for 16S RNA coding gene assignation**

---

title: "Assignation 16S"

author: "Renaud Prevel & Raphael Enaud"

date: '`r format(Sys.time(), "%d %m %Y")`'

header-includes:

- \usepackage{color, fancyvrb}

output:

rmdformats::readthedown:

highlight: kate

numbersections : yes

html_document:

fig_height: 7

fig_width: 10

---

```{r knitrinit, include=FALSE, warning=FALSE, message=FALSE, cache=FALSE}

library(rstudioapi)

setwd(dirname(rstudioapi::getActiveDocumentContext()$path))

load("./assignation.rda")

library(knitr)

library(rmdformats)

library("kableExtra")

library(DESeq2)

library("ggplot2")

library("readxl")

library("dplyr")

library(knitr)

library(rmdformats)

library(psy)

library(Rcpp)

library(nnet)

library(dplyr)

library(ggplot2)

library(psy)

library(prettyR)

library(corrplot)

library(readr)

library(questionr)

library(finalfit)

library(labelled)

library("dada2")

library(ShortRead)

library(Biostrings)

library(DECIPHER)

library("plyr")

theme_set(theme_bw())

library (phyloseq)

library(BiocStyle)

library(ape)

library(tidyr)

library(broom)

library(dplyr)

library(Hmisc)

library (microbiome)

library (picante)

library(plyr)

library(reshape2)

library(doBy)

library(RVAideMemoire)

library (vegan)

library(heatmap.plus)

library(RColorBrewer)

library(gplots)

library(kableExtra)

library(gridExtra)

library(knitr)

library(microbiomeSeq)

library(adespatial)

library(ggpubr)

library (devtools)

library(yingtools2)

library("microDecon")

```

```{r, include=FALSE}

# Creation of the phyloseq object

samdf<- read_xlsx("./Sample Data.xlsx")

samples_df <- sample_data(samdf)

rownames(samples_df) <- samples_df$sample

samples_df$Groupe <- factor(samples_df$Groupe)

samples_df$Sample_or_Control <- factor(samples_df$Sample_or_Control)

phylo <- phyloseq(otu_table(seqtab.nochim, taxa_are_rows=FALSE),

sample_data(samples_df),

tax_table(taxa))

dna <- Biostrings::DNAStringSet(taxa_names(phylo))

names(dna) <- taxa_names(phylo)

phylo <- merge_phyloseq(phylo, dna)

taxa_names(phylo) <- paste0("ASV", seq(ntaxa(phylo)))

phylo

```

# Check the number of reads

```{r, echo=FALSE}

df <- as.data.frame(sample_data(phylo)) # Put sample_data into a ggplot-friendly data.frame

df$LibrarySize <- sample_sums(phylo)

df <- df[order(df$LibrarySize),]

df$Index <- seq(nrow(df))

ggplot(data=df, aes(x=Index, y=LibrarySize, color=Sample_or_Control, label = sample)) + geom_point()

kableExtra::kable(sort(sample_sums(phylo))) %>%

kable_styling() %>%

scroll_box(width = "100%", height = "600px")

```

# Selection of bacterial ASVs

```{r, echo=FALSE}

phylo = subset_taxa(phylo, Kingdom=="Bacteria")

phylo

#Removal of phylum with a single taxa

phylo <- subset_taxa(phylo, !is.na(Phylum) & !Phylum %in% c("","Bdellovibrionota","Chloroflexi","Deinococcota","<NA>","NA"))

phylo

```

# Filtering of minority ASVs

```{r, echo=FALSE}

# ASV present in less than 3 samples

condition <- function(x) { sum(x > 0) >= 2 }

taxaToKeep <- filter_taxa(phylo, condition)

phylo <- prune_taxa(taxaToKeep, phylo)

phylo = prune_samples(names(which(sample_sums(phylo) >= 1)), phylo)

phylo = prune_taxa(names(which(taxa_sums(phylo) >= 1)), phylo)

phylo

```

# Rarefaction curves

```{r, include=FALSE, warning=FALSE, message=FALSE, cache=FALSE, fig.width = 10, fig.height= 10}

library(vegan)

library(ggplot2)

library("ranacapa")

phylo = prune_samples(names(which(sample_sums(phylo) >= 1)), phylo)

rarefactioncurves = ggrare(phylo, step = 100, label = "sample", color = "Sample_or_Control",se = FALSE)

```

```{r, echo=FALSE, warning=FALSE, message=FALSE, cache=FALSE, fig.width = 10, fig.height= 10}

rarefactioncurves

```

# Beta diversity

Measurement of Beta Diversity by Bray Curits

## MDS representation (multi-dimensional scaling)

```{r, include=FALSE, warning=FALSE, message=FALSE, cache=FALSE, fig.width = 10, fig.height= 10}

# Transform data to proportions as appropriate for Bray-Curtis distances

ps.prop <- transform_sample_counts(phylo, function(otu) otu/sum(otu))

ord.nmds.bray <- ordinate(ps.prop, method="MDS", distance="bray")

```

```{r, echo=FALSE, warning=FALSE, message=FALSE, cache=FALSE, fig.width = 10, fig.height= 10}

plot_ordination(ps.prop, ord.nmds.bray, type="samples", label = "sample", color = "Sample_or_Control", title="MDS")

```

## Representation NMDS (Non-metric multi-dimensional scaling)

```{r, include=FALSE, warning=FALSE, message=FALSE, cache=FALSE, fig.width = 10, fig.height= 10}

ord.nmds.bray <- ordinate(ps.prop, method="NMDS", distance="bray")

```

```{r, echo=FALSE, warning=FALSE, message=FALSE, cache=FALSE, fig.width = 10, fig.height= 10}

plot_ordination(ps.prop, ord.nmds.bray, type="samples", label = "sample", color = "Sample_or_Control",title="NMDS")

```

# Beta diversity tree (with Bray Curtis dissimilarity index)

```{r, include=FALSE, warning=FALSE, message=FALSE, cache=FALSE}

library("ape")

random_tree = rtree(ntaxa(phylo), rooted=TRUE, tip.label=taxa_names(phylo))

phylo1 = merge_phyloseq(phylo,random_tree)

phylo1

# Dissimilarity measurement with Bray Cutris

GPUF <- round(distance(phylo1,"bray"),3)

```

```{r, echo=FALSE, warning=FALSE, message=FALSE, cache=FALSE, fig.width = 7, fig.height= 26}

# Creating a tree

## Manually define color-shading vector based on sample type.

colorScale <- rainbow(length(levels(getVariable(phylo1, "Sample_or_Control"))))

cols <- colorScale[getVariable(phylo1, "Sample_or_Control")]

GP.tip.labels <- as(getVariable(phylo1, "sample"), "character")

GP.hclust <- hclust(GPUF, method = "average")

plot(as.phylo(GP.hclust), show.tip.label = TRUE, tip.color = "white")

tiplabels(GP.tip.labels, col = cols, frame = "none",adj = -0.05,cex = 0.7)

```

# Removal of negative controls and Mock

```{r, echo=FALSE, warning=FALSE, message=FALSE, cache=FALSE}

phylo <- subset_samples(phylo, Sample_or_Control =="sample")

phylo = prune_taxa(names(which(taxa_sums(phylo) >= 1)), phylo)

phylo

```

# Resume of the final dataset

```{r, echo=FALSE, warning=FALSE, message=FALSE, cache=FALSE}

summarize_phyloseq(phylo)

```

# saving the global environment

```{r, echo=FALSE, warning=FALSE, message=FALSE, cache=FALSE}

save(phylo, list,file="Phylo pre processed.rda")

```

**Script for ITS2 coding gene assignation**

---

title: "ITS Assignment"

author: "Renaud Prevel & Raphael Enaud"

date: '`r format(Sys.time(), "%d %m %Y")`'

header-includes:

- \usepackage{color, fancyvrb}

output:

rmdformats::readthedown:

highlight: kate

numbersections : yes

html_document:

fig_height: 7

fig_width: 10

---

```{r knitrinit, include=FALSE, warning=FALSE, message=FALSE, cache=TRUE}

# In "path", create a folder "Fastq" with the sequences R1 and R2.

library(knitr)

library(rmdformats)

library("kableExtra")

library(DESeq2)

library("ggplot2")

library("readxl")

library("dplyr")

library(knitr)

library(rmdformats)

library("kableExtra")

library(DESeq2)

library(psy)

library(Rcpp)

library(nnet)

library(dplyr)

library(ggplot2)

library(psy)

library(prettyR)

library(corrplot)

library(readr)

library(questionr)

library(finalfit)

library(labelled)

library("dada2")

library(ShortRead)

library(Biostrings)

library(DECIPHER)

library("plyr")

library("phyloseq")

library("ranacapa")

theme_set(theme_bw())

library(rstudioapi)

setwd(dirname(rstudioapi::getActiveDocumentContext()$path))

path <- file.path(".")

input <-list.dirs(path, full.names = F, recursive = F)

qual <-"./quality/"

outp <-"./output/"

fastq <- "./Fastq"

R1 <- "./R1"

fnFs <- sort(list.files(fastq, pattern = "_R1_001.fastq.gz", full.names = TRUE))

fnRs <- sort(list.files(fastq, pattern = "_R2_001.fastq.gz", full.names = TRUE))

## ITS2 Primers (ITS7F / 3271R):

FWD <- "GTGARTCATCGAATCTTT"

REV <- "GATATGCTTAAGTTCAGCGGGT"

```

```{r, include=FALSE, warning=FALSE, message=FALSE, cache=FALSE}

# Checking the presence and orientation of primers

allOrients <- function(primer) {

# Create all orientations of the input sequence

require(Biostrings)

dna <- DNAString(primer) # The Biostrings works w/ DNAString objects rather than character vectors

orients <- c(Forward = dna, Complement = Biostrings::complement(dna), Reverse = reverse(dna),

RevComp = reverseComplement(dna))

return(sapply(orients, toString)) # Convert back to character vector

}

FWD.orients <- allOrients(FWD)

REV.orients <- allOrients(REV)

FWD.orients

REV.orients

# Pre filtration

fnFs.filtN <- file.path(fastq, "filtN", basename(fnFs)) # Put N-filterd files in filtN/ subdirectory

fnRs.filtN <- file.path(fastq, "filtN", basename(fnRs))

out <- filterAndTrim(fnFs, fnFs.filtN, fnRs, fnRs.filtN, maxN = 0, multithread = TRUE)

primerHits <- function(primer, fn) {

# Counts number of reads in which the primer is found

nhits <- vcountPattern(primer, sread(readFastq(fn)), fixed = FALSE)

return(sum(nhits > 0))

}

```

Number of primers found :

```{r, echo=FALSE, warning=FALSE, message=FALSE, cache=FALSE}

rbind(FWD.ForwardReads = sapply(FWD.orients, primerHits, fn = fnFs.filtN[[1]]),

FWD.ReverseReads = sapply(FWD.orients, primerHits, fn = fnRs.filtN[[1]]),

REV.ForwardReads = sapply(REV.orients, primerHits, fn = fnFs.filtN[[1]]),

REV.ReverseReads = sapply(REV.orients, primerHits, fn = fnRs.filtN[[1]]))

```

# Primer removal

```{r, include=FALSE, warning=FALSE, message=FALSE, cache=FALSE}

cutadapt <- "C:/Users/EnaudR/AppData/Local/Packages/PythonSoftwareFoundation.Python.3.9_qbz5n2kfra8p0/LocalCache/local-packages/Python39/Scripts/cutadapt.exe" # CHANGE ME to location on your machine

R1 <- file.path(path, "R1")

if(!dir.exists(R1)) dir.create(R1)

R2 <- file.path(path, "R2")

if(!dir.exists(R2)) dir.create(R2)

fnFs.cut <- file.path(R1, basename(fnFs))

fnRs.cut <- file.path(R2, basename(fnRs))

FWD.RC <- dada2:::rc(FWD)

REV.RC <- dada2:::rc(REV)

# Trim FWD and the reverse-complement of REV off of R1 (forward reads)

R1.flags <- paste("-g", FWD, "-a", REV.RC)

# Trim REV and the reverse-complement of FWD off of R2 (reverse reads)

R2.flags <- paste("-G", REV, "-A", FWD.RC)

# Run Cutadapt

for(i in seq_along(fnFs)) {

system2(cutadapt, args = c(R1.flags, R2.flags, "-n", 2, # -n 2 required to remove FWD and REV from reads

"-m", 20, "-o", fnFs.cut[i], "-p", fnRs.cut[i], # output files, filtrage des reads au minimum 20 pb

fnFs.filtN[i], fnRs.filtN[i])) # input files

}

```

```{r, echo=FALSE, warning=FALSE, message=FALSE, cache=FALSE}

rbind(FWD.ForwardReads = sapply(FWD.orients, primerHits, fn = fnFs.cut[[1]]),

FWD.ReverseReads = sapply(FWD.orients, primerHits, fn = fnRs.cut[[1]]),

REV.ForwardReads = sapply(REV.orients, primerHits, fn = fnFs.cut[[1]]),

REV.ReverseReads = sapply(REV.orients, primerHits, fn = fnRs.cut[[1]]))

```

```{r, include=FALSE, warning=FALSE, message=FALSE, cache=FALSE}

path <- file.path(".")

input<-list.dirs(path, full.names = F, recursive = F)

qual<-"./quality/"

outp<-"./output/"

R1 = "./R1"

write.fasta.dada<-function(dada2, file){

seqs<-dada2::getSequences(dada2)

hash<-paste0(">",sapply(seqs, openssl::sha1, USE.NAMES = F))

write(c(rbind(hash, seqs)),file)

}

for (R1 in R1){

# List FASTQ files

fq <-list.files(file.path(path,"R1"), pattern="_001.fastq.gz")

# Extract sample names, assuming filenames have format: SAMPLENAME_XXX.fastq

sample.names <- sapply(strsplit(fq, "_"), `[`, 1)

# Add the full path

fq <- file.path(R1, fq)

filt_path <- file.path(qual,"R1") # Place filtered files in subdirectory

dir.create(filt_path, recursive = T)

# Prepare future filenames

filt <- file.path(filt_path, paste0(sample.names, ".filt.fastq.gz"))

out.ee <- ldply(1:length(sample.names),function(i) {

fastqFilter(fn = fq[i],

fout = filt[i],

trimLeft = 0,minLen = 100,

maxN=0, maxEE = 1, rm.phix=TRUE,

compress=TRUE)

})

# Dereplication

derep <- llply(1:length(sample.names), function(i){

derepFastq(filt[i], verbose=TRUE)

})

names(derep)<-sample.names

# Learn errors

err <- learnErrors(derep, multithread=3)

# Get variants

dada_obj<-dada(derep, err=err, multithread = 3)

# Create output directory

#outp_rds<-file.path(outp,R1, "RDS")

#dir.create(outp_rds, recursive = T)

#saveRDS(dada_obj, file = paste0(outp_rds,"/",R1,".RDS"), compress = "gzip")

# Produce Variant Table

seqtab<-makeSequenceTable(dada_obj)

# Remove Chimeras

seqtab.nochim <- removeBimeraDenovo(seqtab, verbose=TRUE)

}

```

# List of samples

```{r, echo=FALSE, warning=FALSE, message=FALSE, cache=FALSE}

sample.names

```

# Checking reading quality profiles

```{r, echo=FALSE, warning=FALSE, message=FALSE, cache=FALSE}

plotQualityProfile(fq[1:2])

```

# Filtering reads

```{r, echo=FALSE, warning=FALSE, message=FALSE, cache=FALSE}

# Place filtered files in filtered/ subdirectory

```

# Learning about error rates

```{r, echo=FALSE, warning=FALSE, message=FALSE, cache=FALSE}

plotErrors(err, nominalQ=TRUE)

```

# Bulding an ASVs table

```{r, echo=FALSE, warning=FALSE, message=FALSE, cache=FALSE}

seqtab <- makeSequenceTable(dada_obj)

dim(seqtab)

```

# Checking the distribution of sequence lengths

```{r, echo=FALSE, warning=FALSE, message=FALSE, cache=FALSE}

options(max.print="500")

table(nchar(getSequences(seqtab)))

```

# Remove chimeras

```{r, echo=FALSE, warning=FALSE, message=FALSE, cache=FALSE}

seqtab.nochim <- removeBimeraDenovo(seqtab, verbose=TRUE)

dim(seqtab.nochim)

sum(seqtab.nochim)/sum(seqtab)

table(nchar(getSequences(seqtab.nochim)))

```

# Summary of the different steps of the pipeline

```{r, include=FALSE, warning=FALSE, message=FALSE, cache=FALSE}

getN <- function(x) sum(getUniques(x))

track <- cbind(out, sapply(dada_obj, getN), rowSums(seqtab.nochim))

## If processing a single sample, remove the sapply calls: e.g. replace sapply(dadaFs, getN) with getN(dadaFs)

colnames(track) <- c("input", "filtered", "denoised", "nonchim")

rownames(track) <- sample.names

```

```{r, echo=FALSE, warning=FALSE, message=FALSE, cache=FALSE}

options(max.print="600")

track

```

# Assignment

```{r, include=FALSE, warning=FALSE, message=FALSE, cache=FALSE}

library(stringr)

unite.ref <- "./sh_general_release_dynamic_s_10.05.2021.fasta" # CHANGE ME to location on your machine

taxa <- assignTaxonomy(seqtab.nochim, unite.ref, multithread = TRUE, tryRC = TRUE)

str_replace_all(taxa, pattern = "k__", replacement = "")

str_replace_all(taxa, pattern = "p__", replacement = "")

str_replace_all(taxa, pattern = "c__", replacement = "")

str_replace_all(taxa, pattern = "o__", replacement = "")

str_replace_all(taxa, pattern = "f__", replacement = "")

str_replace_all(taxa, pattern = "g__", replacement = "")

str_replace_all(taxa, pattern = "s__", replacement = "")

```

Viewing the found assignments :

```{r, echo=FALSE, warning=FALSE, message=FALSE, cache=FALSE}

taxa.print <- taxa # Removing sequence rownames for display only

rownames(taxa.print) <- NULL

head(taxa.print)

```

# saving the global environment

save( out.ee, fq, seqtab, seqtab.nochim, err, track,sample.names, taxa , file = "./assignation Unite.rda")

```
